# Clinical nutrition issues in 2022: What is missing to trust supplemental parenteral nutrition (SPN) in ICU patients?

**DOI:** 10.1186/s13054-022-04157-z

**Published:** 2022-09-10

**Authors:** Mette M. Berger, Rosa Burgos, Michael P. Casaer, Edoardo De Robertis, Juan Carlos Lopez Delgado, Vincent Fraipont, João Gonçalves-Pereira, Claude Pichard, Christian Stoppe

**Affiliations:** 1 Lausanne University Hospital, 1011 Lausanne, Switzerland; 2 University Hospital Vall d’Hebron, Barcelona, Spain; 3 Laboratory and Clinical Department of Intensive Care Medicine, KU Leuven, Leuven, Belgium; 4 Intensive Care Service, Department of Surgical and Biomedical Science, University of Perugia, Perugia, Italy; 5 Area de Vigilancia Intensiva (ICMiD), Hospital Clínic de Barcelona, Barcelona, Spain; 6 Service of Intensive Care Medicine, Citadelle Hospital, Liège, Belgium; 7 Intensive Care Department, Vila Franca de Xira, Portugal; 8 Clinical Nutrition, University Hospital, Geneva, Switzerland; 9 Department of Intensive Care Medicine, Würzburg University, Würzburg, Germany

**Keywords:** Clinical nutrition, Critically ill, Intensive care unit, Nutrition care, Supplemental parenteral nutrition

## Abstract

A multidisciplinary group of international physicians involved in the medical nutrition therapy (MNT) of adult critically ill patients met to discuss the value, role, and open questions regarding supplemental parenteral nutrition (SPN) along with oral or enteral nutrition (EN), particularly in the intensive care unit (ICU) setting. This manuscript summarizes the discussions and results to highlight the importance of SPN as part of a comprehensive approach to MNT in critically ill adults and for researchers to generate new evidence based on well-powered randomized controlled trials (RCTs). The experts agreed on several key points: SPN has shown clinical benefits, resulting in this strategy being included in American and European guidelines. Nevertheless, its use is heterogeneous across European countries, due to the persistence of uncertainties, such as the optimal timing and the risk of overfeeding in absence of indirect calorimetry (IC), which results in divergent opinions and barriers to SPN implementation. Education is also insufficient. The experts agreed on actions needed to increase evidence quality on SPN use in specific patients at a given time point during acute critical illness or recovery.

## Introduction

Over the last decades, critically ill patients have repeatedly been shown to be fed with amounts below the recommendations of the international guidelines. Supplemental parenteral nutrition (SPN) to top-up insufficient enteral nutrition (EN) has been proposed to overcome the building up of large energy deficits: this strategy is included as an option in both the American (ASPEN) and European (ESPEN) clinical nutrition societies’ guidelines [1, 2]. At the same time, the medical community has become aware about the risks of overfeeding [3], the open questions about optimal timing, and the impact of the early endogenous glucose production (EGP) on the energy prescription [4]. The actual high number of uncertainties around SPN result in a limited acceptance and use of the strategy, as confirmed by the EuroPN study: SPN was used in about 10% of nutrition days, generally starting around day 4 [5].

To enable identifying the potential barriers to the prescription of SPN, an international virtual meeting was organized including multidisciplinary participants. An invitation was sent out by Baxter to 20 European experts involved in medical nutrition therapy (MNT), based on their expertise and interest (positive or negative) in the SPN concept expressed during sessions devoted to critically ill patients. Finally, nine participants could attend the meeting and contributed to the discussion: they were physicians managing intensive care units (ICUs) (19–95 beds; average 47). The meeting was followed by two rounds of mail exchange to clarify the text and include the latest relevant publications. Before the meeting, the experts completed a short online survey regarding their respective ICUs and actual nutrition practices, which revealed the inclusion of a broad spectrum of medico-surgical patient types (cardiac surgery, oncology, other major surgery, neurosurgery, burn injuries, trauma, and medical conditions); physicians primarily determined nutrition needs and plans (in acute phases of illness), often using protocols to direct and execute care. Most ICU facilities had a nutrition protocol (7 out of 9). of which six had SPN use included in the protocol, and five had indirect calorimetry (IC) available.

The aim was to discuss the available evidence, and criteria to identify patients who might benefit from its use, and identify the barriers, the hypothesis being that an optimal use of SPN would be associated with an improved clinical outcome.

## Definition of SPN

Hereafter, SPN refers to the administration of parenteral nutrition (PN) when oral and/or EN fails to reach nutrition targets [6–8]. SPN involves a blended approach combining EN with PN to meet patients’ nutrition requirements, based on illness, hemodynamic instability, metabolic measurements, and/or weight-based calculations. Most often industrial solutions containing a predefined mix of carbohydrates, amino acids, and lipids provided as an all-in-one bag are used for this purpose. These bags, as other PN bags, do not contain vitamins and trace elements. The products are generally presented as smaller volumes (500–800 ml).

## Evidence and guidelines

Both ASPEN and ESPEN guidelines mention the use of SPN in critically ill patients while recognizing that there is no strong evidence as to the optimal timing [1, 2]. Based on discussions and guideline recommendations, the experts agreed that more specific criteria are required for initiating SPN. They recommended more evidence; building on the work of Heidegger et al. [9], Ridley et al. [10] and Berger et al. [7], and developing clear protocols to translate guidelines into practice. Recent meta-analysis of these trials suggests clinical benefits with SPN (i.e., lower nosocomial infection and ICU mortality, with improved nutrition intake, and encouraging trends toward functional recovery), which may ultimately improve functional recovery in ICU patients [6, 11].

Moreover, the prospective validation of scores able to identify patients likely to benefit from earlier up-to-target nutrition is lacking. The NUTRIC score appears to be a predictor of overall mortality and indicates potential interactions between nutritional interventions, disease states, and outcomes [2][2]. The NRS score requires further prospective validation [2, 12]. This absence of good scoring option probably reflects the organism’s response to stress with an early EGP, which covers up to 60% of energy needs [4, 13]. Early full feeding is now recognized as non-physiological.

## Potential clinical situations calling for SPN

The authors agreed that inadequate MNT largely depended on the treating physician’s beliefs. Table 1 summarizes the reported key factors driving SPN proposal and prescription.

**Table 1:** Key factors driving SPN prescription in the participating ICU facilities

Persistent hemodynamic instability
Prolonged (> 3–7 days) intolerance to EN, and suspicion of gut hypoperfusion
Patients on ECMO or in prone position who do not tolerate adequate EN for up to 4–7 days
Persistent inability (over several days) to obtain an appropriate enteral access
Hesitancy to increase EN with the thought to minimize or avoid potential complications
Insufficient (< 60–70%) energy and protein delivery via oral or enteral route for 0–7 days (mean just under 4 days)
Failure to reach estimated energy target by ICU Day 4 despite adequate attempts to feed via the enteral route (gastric or post-pyloric)
Pre-existing malnutrition (to prevent further deterioration of nutritional status, always with careful progression over a few days to target)
Hypercatabolism (e.g., burn or cardiac surgery patients who are not meeting energy and protein goals, always with careful progression over a few days to avoid complications)
Prolonged significant vasopressor or inotrope requirements (e.g., patients after complicated cardiac surgery)
Surgeon requests in the immediate postoperative period not to use the intestine
Growing cumulative energy deficit of 3000–6000 kcal, and beyond 10,000 kcal
Suspicion of intestinal ischemia and elevated intraabdominal pressure

*Abbreviations: ECMO* extracorporeal membrane oxygenation, *EN* enteral nutrition, *ICU* intensive care unit

## Barriers to SPN use

The identified barriers to SPN use vary across ICUs and include limited evidence on SPN benefits; unavailability of protocols for therapy initiation; lack of ownership of nutrition care plan by a specific person or discipline; absence of qualified dietitians in ICUs; insufficient time and/or understanding of techniques, including vascular access and SPN prescription; unavailability in various medical centers of appropriate ready-to-use formulations; concerns about infection risk, cost, and staff workload; unavailability of trained prescribers on weekends/holidays; inability to accurately assess energy requirements; uncertainty about the percentage of measured resting energy to be provided at different time points during acute disease and recovery; and lack of understanding regarding the importance of cumulative energy deficit [11, 14].

## Gaps in evidence and preferred outcome variables to establish benefit

Gaps identified in available evidence for SPN use included: uncertainty about the appropriate day to initiate SPN, the day count becoming even more complicated if patients are transferred to the ICU from the hospital ward; exclusion of patients with severe illness and/or pre-existing conditions from clinical trials; concerns about the effect of the endogenous energy production on nutrition requirements early in critical care; inability to define specific outcomes possibly attributable to SPN use.

The experts stressed the importance of including in the trials the functional outcomes, such as time to complete a 6-min walk, handgrip strength measurements, frailty scores, and measures of functional status. Body composition measured with ultrasound, bioelectrical impedance (BIA) and phase angle calculation are practical tools and should be included (Table 2). However, the experts acknowledged that cost, data availability, and data reliability were barriers to collecting functional outcome data, particularly if collected longitudinally. Moreover, assessment of functional outcomes in critically ill patients is complicated by the competing event of death, eliminating the weakest patients from assessment and, thereby, generating a potential source of informal censoring [15–17].

**Table 2:** Summary of essential “optimizers” of SPN in clinical practice and future trials identified during the meeting

Optimizers of outcome while using SPN	Endpoints to be include in trials
Education in nutrition of the critically ill Development of precise protocols translating guidelines to clinical practice Validation of scores able to identify patients likely to benefit from SPN Availability of indirect calorimetry List of variables to monitor during SPN Protocol describing weaning from SPN to EN/oral Further trials adequately designed and powered to generate reliable estimates of treatments effect of SPN on long-term functional outcomes in specific patients at a given time point in disease	Long-term outcome (≥ 90 days) Muscle mass, bioimpedance analysis, phase angle Functional outcomes (handgrip strength walking distance, SF-36) Repeated indirect calorimetry over time Measure of endogenous glucose production Focus on the right dosing and timing of SPN initiation Complications

## Potential role of IC in SPN prescription

The experts suggested that use of IC as a metabolic monitor is an important part of precision medicine, along with regular glucose monitoring, and may reassure clinicians fearful of overfeeding patients while initiating SPN.

But while large RCTs powered for patient-centered outcomes are looked forward to, they may not be able to achieve precision medicine with a personalized approach due to center practice variability, use of equations to set energy targets, and unavailability of IC, which would obscure results. This concern was demonstrated in the TICACOS international RCT, which involved expert centers using IC-guided SPN and was stopped due to slow recruitment [17]. Nevertheless, two recent meta-analyses point in the direction of benefits of a personalized approach based on IC, but these data remain to be further confirmed [18, 19].

## Targeted, concise education about when and how to use SPN is needed

The nutrition care team must be aware of SPN indications and aligned on protocols for initiating and monitoring SPN therapy. A combination of evidence-based guidance and expert knowledge is needed to evaluate the needs of an individual patient. The gastrointestinal (GI) tract must be systematically assessed using a validated score, such as the Gastrointestinal Dysfunction Score (GIDS) [20]. Ideally, EN is initiated first and optimized (via the use of prokinetics, a bowel regimen, and intensive monitoring). If intolerance develops, SPN may be combined with EN, or PN may completely replace EN in cases of severe EN intolerance.

## Conclusion

SPN is currently underutilized or used without proper monitoring. More evidence supporting the need for, and outcomes of SPN use is needed as treating each patient as an individual is critical. The evidence must focus on the optimal timing of SPN initiation in specific patients (based on scores or biomarkers) and the correct initial and subsequent dosing, to improve clinical outcomes. The participants agreed to the need of appropriate clinical trials of adequate size are required to detect potential clinical meaningful effects [11]: functional outcomes may not only be more patient-centered but also more likely to capture specific benefits of MNT [11]. Future trials must assess and report on nutrition risks and complications. The importance of including functional outcomes in future clinical trials was reiterated.

Therefore, the group proposes a three-pronged call to action and strategy. First, rigorously designed clinical trials with outcomes focused on the right dosing and timing of SPN initiation are needed. Second, education and awareness of the importance of optimal MNT for better patient outcomes are crucial. Finally, clinical protocols translating guidelines into practice are needed. Clinical protocols will facilitate the adoption of an effective approach to SPN use, along with (as appropriate) oral nutrition and EN. These steps are likely to improve outcomes in ICU patients. This paper is a call to action to focus research on SPN as a complementary or “blended” therapy to EN and to understand that MNT is not “either PN or EN.” Both may often be needed to attain optimal nutritional status.

## Data Availability

Not applicable.
